# CD146 promotes metastasis and predicts poor prognosis of hepatocellular carcinoma

**DOI:** 10.1186/s13046-016-0313-3

**Published:** 2016-02-29

**Authors:** Guoqing Jiang, Long Zhang, Qin Zhu, Dousheng Bai, Chuanyong Zhang, Xuehao Wang

**Affiliations:** Department of Hepatobiliary Surgery, Clinical Medical College of Yangzhou University, Yangzhou, P.R. China; Key Laboratory of Living Donor Liver Transplantation, Ministry of Public Health; Department of Liver Transplantation Center, The First Affiliated Hospital of Nanjing Medical University, Nanjing, P.R. China

**Keywords:** Hepatocellular carcinoma, Metastasis, Recurrence, EMT, CD146

## Abstract

**Background:**

Hepatocellular carcinoma (HCC) is the third leading cause of cancer-related mortality worldwide. Recurrence and metastasis after curative resection remain critical obstacles in HCC treatment. CD146 predicted poor prognosis of a variety of cancers including melanoma, breast tumors, prostate cancer, and gastric cancer. However, the role of CD146 in HCC has not yet been systematically explored.

**Methods:**

To investigate the role of CD146 in HCC, we evaluated its expression in HCC tissues and HCC cell lines using real-time PCR and western blotting (WB). Second, we established HCC cell lines that stably overexpressed and interfered CD146 and explored the function of CD146 in HCC in vitro and in vivo. Third, we conducted microarray analysis to investigate the potential mechanism by identifying differentially expressed genes. Last, follow ups were conducted to help uncover the connection of CD146 expression and the prognosis of HCC patients.

**Results:**

We found that CD146 was overexpressed in HCC tissues and that high CD146 expression predicted poor overall survival time and shorter recurrence period in HCC patients. In vitro and in vivo experiments indicated that CD146 promoted migration and invasion of HCC cell lines. Further study indicated that CD146 promoted epithelial mesenchymal transition (EMT), IL-8 upregulation, and STAT1 downregulation. CD146 was upregulated in HCC tissues and cell lines.

**Conclusions:**

CD146 promoted metastasis of HCC cells and predicted poor prognosis of HCC patients. CD146 induced EMT, and IL-8 upregulation and STAT1 downregulation may be the potential underlying mechanism. The exact mechanism still needs further investigation.

**Electronic supplementary material:**

The online version of this article (doi:10.1186/s13046-016-0313-3) contains supplementary material, which is available to authorized users.

## Background

Hepatocellular carcinoma (HCC) is the fifth most common malignant cancer and the third leading cause of cancer-related mortality in China [1]. Recurrence and metastasis after curative resection are the most challenging burdens for HCC treatment [2]. However, the underlying mechanism of HCC metastasis is largely unknown.

CD146 was first reported in malignant melanomas [3]. CD146 is a 113-kD membrane glycoprotein that contains five immunoglobulin-like domains, a transmembrane region, and a short cytoplasmic tail [4]. Early research indicated that CD146 expressed in normal tissues is restricted to the blood vessels and smooth muscle cells [5]. Whereas, subsequent research indicated CD146 is a multifunctional molecule that participates in several physiological and pathological processes involving in development, immunity, and angiogenesis [6]. CD146 mediates development of the nervous system, kidney, and retina [7–9]. Knockdown of CD146 protein expression hinders vascular development whereas overexpression of CD146 in zebrafish induces sprouting angiogenesis [10]. CD146 was also found to play a critical role in cancer progression. In most cancers, CD146 was found to promote cancer progression, enhanced migration and invasion was observed in melanoma, gallbladder adenocarcinoma, breast cancer and prostate cancer [11–14]. In gastric cancer, lung adenocarcinoma, malignant pleural mesothelioma, and non-small-cell lung cancer, CD146 has been identified as an indicator of poor prognosis [15–18]. However, in oral mucoepidermoid carcinoma, CD146 expression was greater in intermediate/high grade tumors, was weaker in patients that presented local recurrence, regional and distant metastasis [19]. Most researches above focused on relationship between CD146 expression and clinical outcomes, the underlying mechanism of CD146 induced migration and invasion has not yet been systemically explored. the mechanism besides CD146 induced angiogenesis was rarely explored. In HCC research, CD146 was reported to be used as endothelial marker in selective targeting of liver cancer treatment [20]. The role of CD146 in HCC cells has not yet been explored.

In this study, we set out to investigate the role of CD146 in HCC. We mainly focused on the CD146 on tumor cells whereas the role of CD146 in endothelial cells was not addressed. Firstly, the expression of CD146 in HCC tissues and HCC cell lines was detected using RT-PCR and WB, the location of CD146 was confirmed using IHC; Secondly, we established in vitro and in vivo models to investigate the biologic function of CD146 in HCC cells; Thirdly, we conducted a mRNA microarray to systemically explore the mechanism of CD146 induced biologic behavior variation of HCC cells; lastly, CD146 expression and clinical prognosis of HCC patients was concerned.

## Methods

### Cell lines and cell culture

Cell lines used in this study include eight human HCC cell lines, MHCC-97H, MHCC-97 L, HepG2, SMMC-7721, Focus, YY-8103, LM3, and HLF, and one human noncancerous hepatic cell line, L02. All these cell lines were provided and identified by our key laboratory of living donor liver transplantation. All cells were cultured in Dulbecco’s Modified Eagle medium (Gibco, Grand Island, NY, USA) containing 10 % FBS (Hyclone, Logan, UT, USA), 100 U/mL penicillin and 100 μg/mL streptomycin (Invitrogen, Carlsbad, CA, USA) at 37 °C in 5 % CO_2_.

### Patients and follow-up

A total of 120 HCC samples and homologous noncancerous tissue samples were randomly obtained from the samples that were removed from the patients and stored in the department of pathology between June 2008 and May 2010, snap frozen in liquid nitrogen, and stored at −80 °C until use. All samples were subjected to routine pathological examination at the First Affiliated Hospital of Nanjing Medical University. All patients underwent curative resection of HCC at the First Affiliated Hospital of Nanjing Medical University (Nanjing, China). The patients’ age ranged from 31 to 83 years. Detailed clinicopathological characteristics of the 120 HCC patients are listed in Table 1. Written informed consent was obtained from each patient before surgery. The patients were followed up after surgical treatment until August 2014. This study was approved by the Ethics Committee of the First Affiliated Hospital of Nanjing Medical University.

**Table 1:** Clinical characteristics of HCC patients and correlation with CD146 level

Clinical characteristics	Patients (*n* = 120)	CD146 High	CD146 low	*P* value
Age(Y)	≥ 50	41	42	
	< 50	17	20	0.727
Sex	Male	52	54	
	Female	6	8	0.663
HBsAg	Positive	51	60	
	Negative	7	2	0.136^a^
Child-Pugh score	A	56	61	
	B	2	1	0.953^a^
Liver cirrhosis	Yes	51	57	
	No	7	5	0.465
AFP (ng/ml)	≥ 400	39	27	
	< 400	19	35	0.009
Tumor number	single	45	51	
	multiple	13	11	0.523
Tumor size (cm)	≥ 5	42	43	
	< 5	16	19	0.713
Vascular invasion	Yes	24	13	
	No	34	49	0.016
Satellite lesion	Yes	13	5	
	No	45	57	0.028
Tumor encapsulation	Complete	27	42	
	None	31	20	0.019
Edmondson stage	I-II	20	37	
	III-IV	38	25	0.006
Recurrence	Yes	47	23	
	No	11	39	<0.001

*Abbreviations: HBsAg* Hepatitis B surface antigen, *AFP* α-fetoprotein, *HCC* hepatocellular carcinoma

^a^Continuous correction

### RNA extraction and quantitative RT-PCR

Total RNA was extracted from tissue samples and cell lines using Trizol Reagent (Takara, Dalian, China). RNA concentrations and quality were determined with a NanoDrop ND-2000 spectrophotometer (NanoDrop Technologies, IL, USA) and gel analysis. Reverse transcription was performed using PrimeScript® RT Reagent Kit (Takara). Real-time polymerase chain reaction (RT-PCR) amplifications were carried out on ABI stepone RT-PCR Detection System (ABI, Carlsbad, CA, USA) using the SYBR Green PCR detection kit (Takara) according to the manufacturer’s introductions. Primers are summarized in Table S1 (Additional file 1). The cycle time (Ct) values of the selected genes were first normalized with the value of beta actin of the same sample, and then the relative expression of each gene was analyzed using the 2-ΔΔCt method.

### Western blotting

Western blotting was performed using standard techniques as previously described [11]. Briefly, total protein was extracted using RIPA lysis buffer (50 mM Tris-Cl pH 7.4, 150 mM NaCl, 0.5 % sodium deoxycholate, 1 % NP-40, 0.1 % SDS, 1 mM EDTA, 100 mM NaF, 1 mM Na3VO4, 1 mM PMSF, and 2 μg/mL aprotinin) on ice. Protein samples (50 μg) were separated by sodium dodecyl sulfate-polyacrylamide gel electrophoresis and transferred to polyvinylidene difluoride membranes. Membranes were blocked with 5 % nonfat milk in TBST (10 mM Tris, pH 7.4, 150 mM NaCl, and 0.1 % Tween-20) at room temperature for 1 h and incubated with indicated primary antibodies at 4 °C overnight with gentle rocking. After washing with TBST, the membrane was incubated with the appropriate HRP-conjugated secondary antibodies for 1 h at 37 °C. After extensive washing with TBST, proteins were visualized by the enhanced chemiluminescence detection kit in accordance with the manufacturer’s recommendations (Thermo Fisher scientific, MA , USA). Antibodies used in this study are summarized in Table S2 (Additional file 2).

### Immunohistochemistry

Immunohistochemical staining was performed using the diaminobenzidine detection kit (Maixin-Bio, Fuzhou, China) following the manufacturer’s instructions. Briefly, human hepatocellular carcinoma tissues with matched peritumor tissues were processed by standard histological techniques. Tissue sections were deparaffinized and rehydrated. Then, sections were heated in a pressure cooker for 2 min to repair antigenicity and were treated with 3 % H_2_O_2_ for 10 min to inactivate endogenous peroxidase activity and incubated with goat serum for 10 min to block nonspecific antibody binding. Sections were incubated with a primary antibody at 37 °C for 1 h, a biotin-labeled secondary antibody for 10 min, and streptavidin-peroxidase conjugate for 10 min. A solution of 0.02 % iaminobenzidine was used as chromogen to visualize peroxidase activity. The sections were lightly counterstained with hematoxylin, mounted with Permount, and examined by light microscopy.

### Establishment of stable cell clones

Lentiviruses overexpressing CD146 (Lv-CD146) or silencing CD146 (Lv-shCD146) were purchased from Genechem (Shanghai, China). The sequence of interfere chain was listed in Table S3 (Additional file 3). Lentivirus was infected into HCC cells with a multiplicity of infection (MOI) ranging from 5 to 20 in the presence of polybrene (10 μg/mL). Low CD146 expressing cell lines MHCC-97 L and HLF were infected with Lv-CD146, defined as 97 L-CD146 and HLF-CD146. High CD146 expressing cell lines MHCC-97H and SMMC-7721 were infected with Lv-shCD146, defined as 97H-shCD146 and 7721-sh-CD146. At 72 h after infection, cells were selected for 1 week using puromycin (5 μg/mL). The selected cell lines were prepared for subsequent experiments.

### Transwell cell migration and invasion assays

Transwell chambers with an 8-μm pore size were used to measure migration and invasion ability of HCC cells. For migration assays, 5 × 10^4^ cells in 250 μL DMEM containing 0.2 % FBS were seeded into the upper chamber and 500 μL DMEM containing 10 % FBS was added to the lower chamber. For invasion assays, the upper chamber was pre-coated with 50 μL 1:1 mixture of Matrigel (BD Biosciences, USA) and DMEM overnight before 1 × 10^5^ cells were seeded. Cells were incubated in 5 % CO_2_ at 37 °C for 24 h (migration) or 72 h (invasion), Then, cells on the upper side of well were removed, the wells were fixed in methanol for 20 min, and crystal violet was used to stain cells. Photographs of five random fields were captured for quantification analysis. Three identical replicates were performed.

### Animals

Male nude mice (4–6 weeks old) were purchased from Yangzhou University and housed in Nanjing Agricultural University under specific pathogen-free conditions and cared for according to the institutional guidelines for animal care. All animal experiments met the National Institutes of Health guidelines and were approved by the Committee on the Ethics of Animal Experiments of Nanjing Medical University.

### In vivo metastasis assays

A total of 5 × 10^6^ tumor cells were suspended in 100 μL DMEM and then injected subcutaneously into the upper right flank region of nude mice. When the subcutaneous tumor reached approximately 1 cm in length (approximately 2–4 weeks after injection), it was removed, minced into small pieces of equal volume (1 mm^3^), and implanted into the livers of nude mice (10 per group). All mice were monitored once every 3 days, and six mice of each group were sacrificed 6 weeks later. The liver and lung tissues were dissected, fixed with 4 % phosphate-buffered neutral formalin, and prepared for histological examination. All metastatic foci in the lung were calculated microscopically to evaluate the development of pulmonary metastasis.

### Microarray processing and analysis

Total RNA was isolated from HLF-vec (*n* = 3) and HLF-CD146 (*n* = 3) cell lines. RNA samples were analyzed by microarray expression profiling using the Affymetrix Human GeneChip primeview (Affymetrix) according to the manufacturer’s instructions. Briefly, cDNA target preparation and in vitro transcription were conducted using the GeneChip 3′IVT PLUS Kit. Arrays were washed, stained, and processed using the GeneChip Hybridization Wash and Stain Kit, after which they were imaged using the Affymetrix GeneChip Scanner 3000 for subsequent generation of raw data. Genes significantly differentially expressed between HLF-vec and HLF-CD146 cells were selected based on fold change >1.5 and *P* < 0.05. Functional pathway analysis was conducted using KEGG pathway enrichment analysis and gene ontology analysis according to the manufacturer’s instructions.

### Statistical analysis

Statistical analyses were performed using SPSS 18.0 (SPSS, Chicago, IL, USA) or Prism 5.0 (GraphPad Software, La Jolla, CA, USA) software. The results were presented as the mean ± standard error of mean. Quantitative data were performed by the two-tailed Student’s t-test. Categorical data were analyzed by χ^2^ test. Kaplan–Meier and log-rank analysis was used to assess the survival between subgroups. *P* < 0.05 was considered statistically significant.

## Results

### The expression of CD146 is upregulated in HCC

**Fig. 1:**
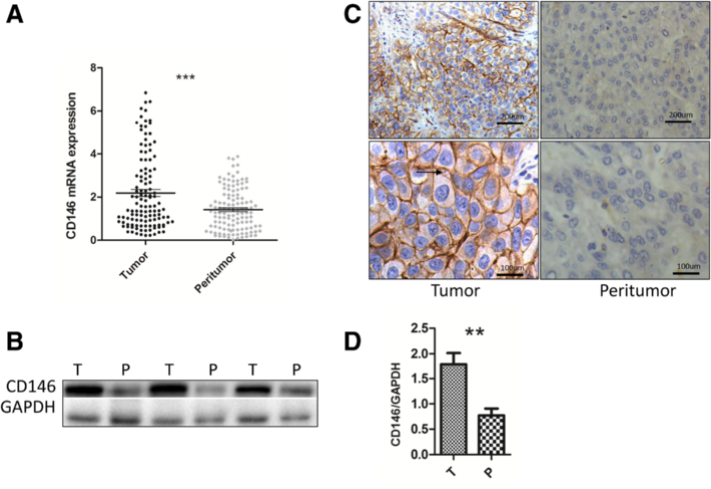
CD146 up-regulated in HCC Patients. **a** RT-PCR of 120 paired HCC and peritumor tissues (***P < 0.001). **b** Western Blotting of 3 paired HCC and peritumor tissues. **c** IHC of the location of CD146 is expressed on the membrane of HCC cells (*black arrow*). **d** Gray scale analysis of averaged CD146/GAPDH in **b** (*n* = 3,**P < 0.01)

**Fig. 2:**
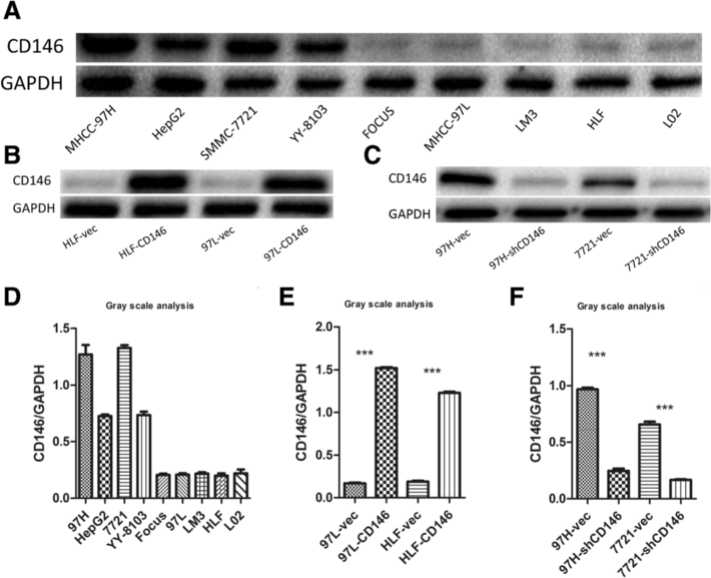
Construction of HCC cell lines stably overexpress or interfere CD146 expression. **a** Western Blot analysis of CD146 in HCC cell lines. **b** Construction of high CD146 expression cell lines. **c** Construction of low CD146 expression cell lines. **d** Gray scale analysis of CD146/GAPDH in **a** (*n* = 3). **e** Gray scale analysis of CD146/GAPDH in B (*n* = 3, ***P < 0.001). **f** Gray scale analysis of CD146/GAPDH in **c** (*n* = 3, ***P < 0.001)

To investigate the role of CD146 in HCC, we first explored the expression of CD146 in HCC tissues and HCC cell lines. We examined CD146 mRNA levels in 120 paired HCC and peritumoral samples using RT-PCR and found that the average expression level of CD146 was significantly higher in HCC than that in peritumoral tissues (Fig. 1a). The upregulation of CD146 was confirmed in 20 paired samples randomly obtained from the 120 paired HCC samples using WB (Fig. 1b (3 pairs) and d, and Additional file 4: Figure S1 (17 pairs)). Immunohistochemical assays revealed that CD146 was located on the membrane of HCC cells (Fig. 1c). CD146 expression was also observed in vascular endothelial cells with much lower intensity, no expression was observed in normal liver cells or other cirrhotic tissue. We then performed RT-PCR and WB to investigate the expression of CD146 in HCC cell lines and noncancerous liver cell lines. We found that the expression of CD146 was markedly upregulated in several HCC cell lines compared with noncancerous cell lines (Fig. 2a and d). Taken together, these data indicated that CD146 is expressed in HCC cells, the expression of CD146 is upregulated in HCC.

### CD146 promotes migration and invasion of HCC cell lines

**Fig. 3:**
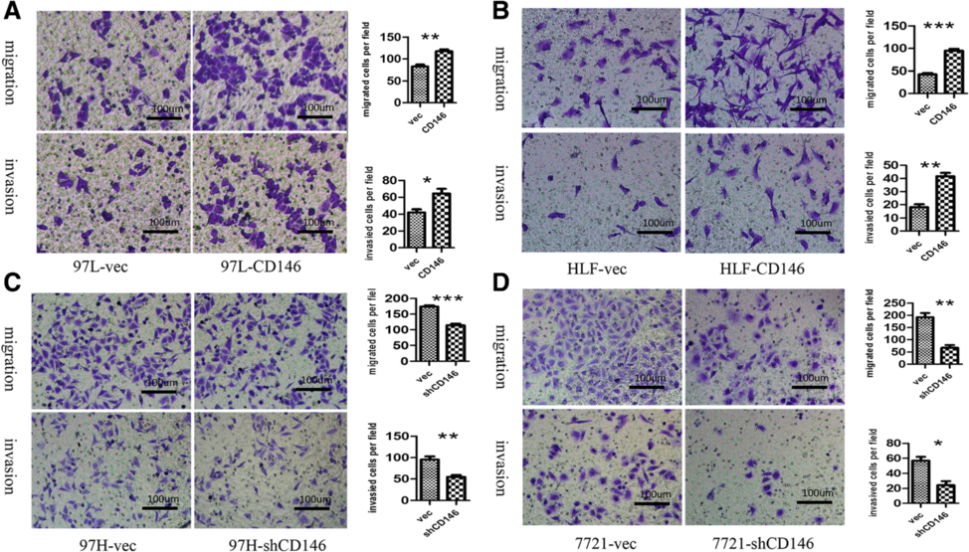
In vitro migration and invasion assays. **a** Overexpression of CD146 in 97 L enhanced cell migration and invasion in transwell assays (*n* = 3, *P < 0.05, **P < 0.01). **b** Overexpression of CD146 in HLF enhanced cell migration and invasion in transwell assays (*n* = 3, **P < 0.01, ***P < 0.001). **c** Inhibition of CD146 in 97H decreased cell migration and invasion in transwell assays (*n* = 3, **P < 0.01, ***P < 0.001). **d** Inhibition of CD146 in SMMC-7721 decreased cell migration and invasion in transwell assays (*n* = 3, *P < 0.05, **P < 0.01)

To determine the biological function of CD146 in HCC, we first established HCC cell lines that stably overexpress or repress CD146 expression using lentivirus as a vehicle. We overexpressed CD146 in 97 L and HLF cells, which are low CD146-expressing cell lines, by lentiviral particles that overexpresses CD146 and renamed these cell lines as 97 L-CD146 and HLF-CD146 (Fig. 2b and e). We also knocked down CD146 in 97H and SMMC7721 cells, which are high CD146-expressing cell lines, using lentiviral particles that expresses CD146 shRNA, and renamed these cells as 97H-shCD146 and SMMC7721-shCD146 (Fig. 2c and f). We then assessed the effects of CD146 on cell growth, migration, invasion, apoptosis and cell cycle. No obvious differences were observed in cell growth (Additional file 5: Figure S2), apoptosis, or cell cycle (Additional file 6: Figure S3) among all the experimental cells. However, transwell assays demonstrated that upregulation of CD146 expression significantly enhanced the migration and invasion capacities of 97 L-CD146 (Fig. 3a) and HLF-CD146 cells (Fig. 3b). Consistent with these data, CD146 repression in 97H-shCD146 (Fig. 3c) and SMMC7721-shCD146 cells (Fig. 3d) significantly reduced cell migration and invasion. These results indicated that CD146 promotes migration and invasion of HCC cells in vitro.

**Fig. 4:**
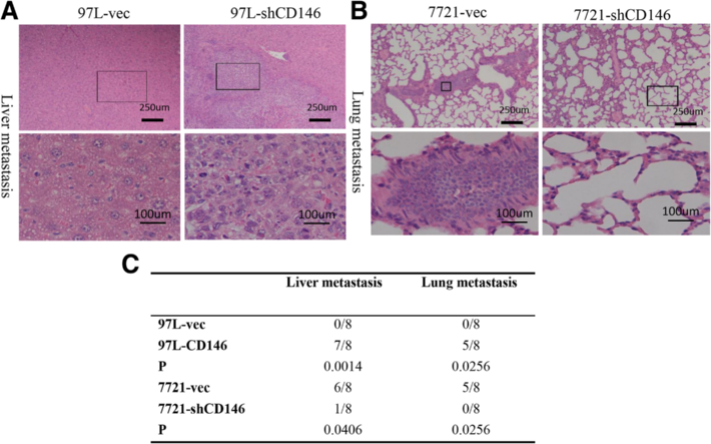
In vivo metastasis assays using orthotropic tumor metastasis model. **a** HCC cell lines with high CD146 indicated more frequent metastasis nodules in liver (40X vs 200X). **b** HCC cell lines with high CD146 indicated more frequent metastasis nodules in lung (40X vs 200X). **c** statistical analysis of liver and lung metastasis

We further examined the role of CD146 in HCC metastasis by establishing an orthotropic tumor metastasis model in nude mice, which closely mimics the process of human HCC metastasis after the formation of the primary foci. Histological analysis of liver tissues revealed a significant effect of CD146 in local invasion of the HCC tumors in the livers. The tumors of the high-CD146 expressing HCC groups (97H-vec and 7721-CD146) had more frequent invasive growth fronts with irregular tumor borders, whereas those of the low-CD146 expressing HCC groups (7721-vec and 97H-shCD146) showed less invasive foci and regular tumor borders. Histological analysis of lung tissue showed that the incidence of lung metastasis in the high CD146-expressing HCC groups was significantly higher than that in the low CD146-expressing HCC groups (Fig. 4). Taken together, these data confirmed that CD146 promotes migration and invasion of HCC cell lines.

### CD146 promotes EMT and IL-8 upregulation and STAT1 downregulation

**Fig. 5:**
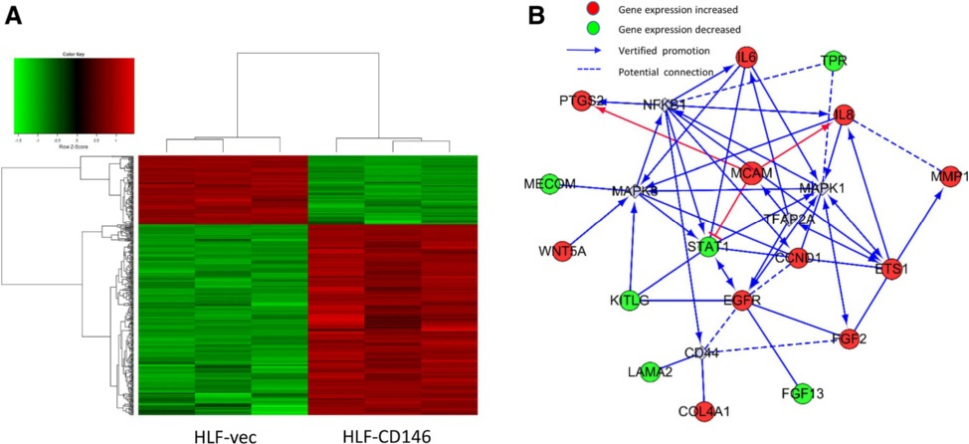
Mechanism analysis of CD146 induced metastasis. **a** A 3 × 3 DNA microarray of HLF-vec and HLF-CD146. A total of 496 genes were found modified after CD146 overexpression (*P* < 0.05 and fold change >1.5). **b** GO analysis of potential regulatory mechanisms of CD146

To uncover the mechanism underlying CD146-induced migration and invasion, we conducted a microarray analysis to identify genes whose expression was modified by CD146 upregulation using HLF-vec and HLF-CD146 cell lines (Fig. 5a). A total of 131 genes were upregulated and 365 genes were downregulated after CD146 overexpression (*P* < 0.05 and fold change >1.5). Supervised analysis was conducted following KEGG and the Gene Ontology technical route. We focused on cancer-related pathways and found that 16 genes showed modified expression upon CD146 overexpression (Additional file 7: Table S4). A network diagram was drawn to show the potential connection between each gene (Fig. 5b).

**Fig. 6:**
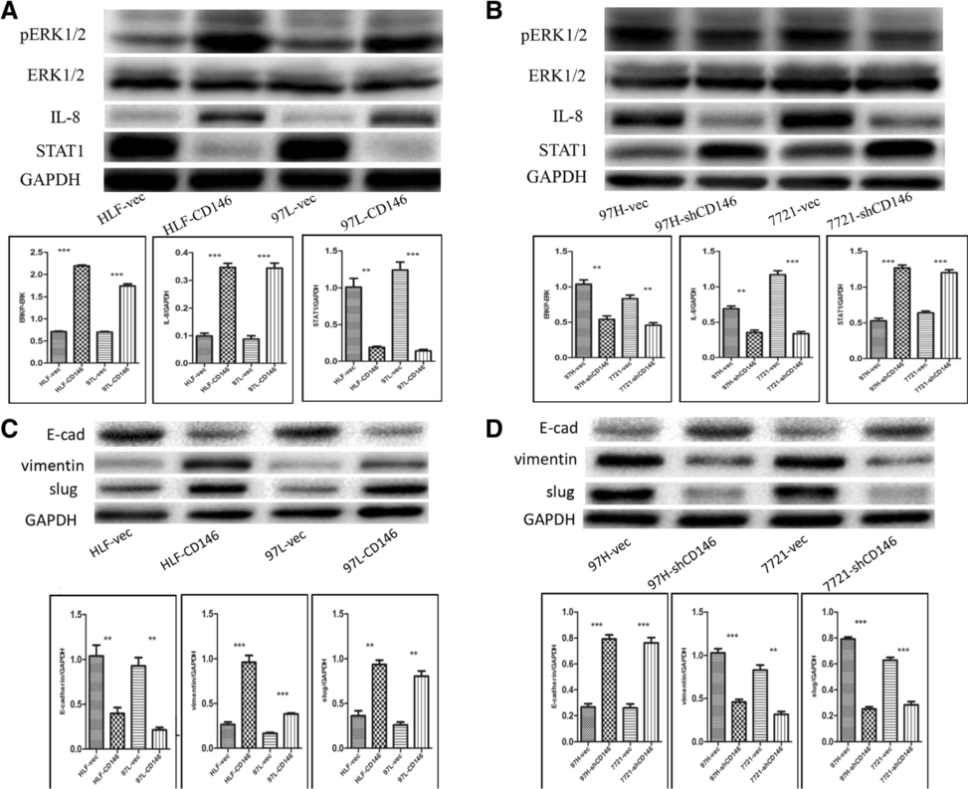
Western blotting vertification of the mechanism of CD146 induced metastasis. **a** CD146 induced ERK1/2 phosphorylation, IL-8 upregulation and STAT1 down regulation in HLF and 97 L cell lines (*n* = 3, **P < 0.01, ***P < 0.001). **b** Interfere CD146 inhibited ERK1/2 phosphorylation, induced IL-8 downregulation and STAT1 upregulation in 97H and 7721 cell lines (*n* = 3, **P < 0.01, ***P < 0.001). **c** CD146 induced EMT (E-cad downregulation, vimentin, slug upregulation ) in HLF and 97 L cell lines (*n* = 3, **P < 0.01, ***P < 0.001). **d** Interfere CD146 inhibited EMT in 97H and 7721 cell lines (*n* = 3, **P < 0.01, ***P < 0.001)

Further verification was conducted using western blotting. Interlukin-8 (IL-8) was upregulated whereas signal transducer and activator of transcription 1 (STAT1) was downregulated after CD146 overexpression (Fig. 6a) and the opposite effects were observed upon CD146 downregulation (Fig. 6b). Pathway analysis indicated that mitogen activated protein kinase 1 (MAPK1) may be the potential pathway by which CD146 induced IL-8 and STAT1 modification. We confirmed that phosphorylation of MAPK1 [Alternative Name: extracellular signal regulated kinase 1 and 2 (ERK1/2)] was markedly increased after CD146 overexpression (Fig. 6a) and decreased after CD146 downregulation (Fig. 6b).

We also tested migration-associated EMT markers and found that E-cadherin was downregulated and vimentin and slug were upregulated in the CD146 overexpressed HCC cells (Fig. 6c). Interfering with CD146 expression showed the opposite results (Fig. 6d).

Taken together, these data indicated that CD146 may regulate IL-8 to activate the MAPK1 signaling pathway, and further regulate STAT1 and EMT to promote migration and invasion of HCC cell lines.

### CD146 predicted poor prognosis of HCC patients

We also followed up HCC patients in this study. 120 patients were divided into CD146 high group (*n* = 58) and CD146 low group (*n* = 62) according to their relative CD146 mRNA expression, averaged 2^-ΔCT value was used as a cut off point. Patient demographics were listed in Table 1. By the time of analysis, recurrence had occurred in 70 of 120 patients, with a mean follow-up time of 38.0 ± 2.1 months (median, 23.0 months; range, 6.0–73.0 months). Among these 70 patients, 58 had intrahepatic recurrence only, 5 had lung metastasis only, and 7 suffered both intrahepatic recurrence and lung metastasis.

Recurrence was observed in 43 of 70 patients with high CD146, while only 27 of 73 patients with low CD146. Among 12 patients who developed lung metastasis, 9 patients had high CD146. In addition, CD146 high group were more likely to manifest high AFP level (*P* = 0.009), vascular invasion (*P* = 0.016), satellite lesions (*P* = 0.028), no tumor encapsulation (*P* = 0.019), poor tumor differentiation (*P* = 0.006), and higher recurrence (*P* < 0.001, Table 1).

**Fig. 7:**
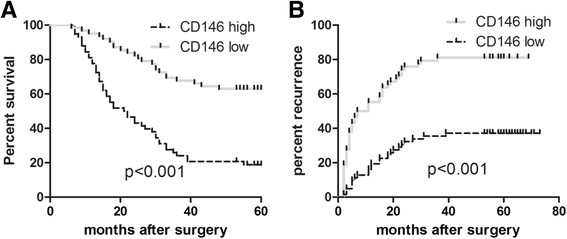
CD146 predicted poor prognosis of HCC patients. **a** Patients in comparative CD146 high group (*n* = 62) had higher recurrence rate than those in comparative CD146 low group (*n* = 58) (*P* <0.001). **b** patients in comparative CD146 high group (*n* = 62) had lower overall survival time than those in comparative CD146 low group (*n* = 58) (*P* < 0.001)

Using a averaged 2^-ΔCT value of CD146 mRNA expression as the cutoff value in univariate analysis, preoperative CD146 mRNA expression showed prognostic significance for TTR (time to recurrence) (*P* = 0.001, Table 2). Patients in CD146 high group had significantly shorter TTR (median, 9.0 months vs. not reached) and higher recurrence rates (81.0 % vs. 37.1 %) than those in Patients in CD146 low group (*P* < 0.001, Fig. 7a). Levels of AFP, tumor number, vascular invasion, satellite lesion, tumor encapsulation and Edmondson stage were also unfavorable prognostic variables for recurrence (all *P* values of <0.05, Table 2). In multivariate analysis, high CD146 mRNA expression was the strongest independent prognostic factor for TTR [hazard ratio (HR) =2.49; 95 % confidence interval (CI), 2.43–4.00, *P* < 0.001; Table 2].

**Table 2:** Univariate and multivariate Cox proportional regression analysis of factors associated with recurrence

Variables	Univariate analysis		Multivariate analysis	
	HR (95 % CI)	*P*	HR (95 % CI)	*P*
Age (> 50yvs. ≤ 50y)	0.90 (0.55–1.48)	0.673	N.A.	N.A.
Sex (male vs. female)	1.01 (0.48–2.10)	0.990	N.A.	N.A.
HBsAg (Positive vs. Negative)	0.77 (0.33–1.77)	0.536	N.A.	N.A.
Child-Pugh score (A vs. B)	1.12 (0.27–4.55)	0.879	N.A.	N.A.
Liver cirrhosis (Yes vs. No)	0.99 (0.45–2.16)	0.977	N.A.	N.A.
AFP (> 400 ng/ml vs. ≤ 400 ng/ml)	1.73 (1.06–2.81)	0.027	1.30 (0.78–2.16)	0.309
Tumor number (Multiple vs. Single)	1.77 (1.04–3.00)	0.022	1.42 (0.82–2.48)	0.214
Tumor size (> 5 cm vs. ≤ 5 cm)	1.35 (0.79–2.31)	0.273	N.A.	N.A.
Vascular invasion (Yes vs. No)	2.16 (1.34–3.48)	0.002	1.14 (0.68–1.93)	0.621
Satellite lesion (Yes vs.No)	2.92 (1.66–5.16)	0.001	1.96 (1.06–3.63)	0.032
Tumor encapsulation (Yes vs. No)	2.26 (1.40–3.63)	0.001	1.64 (0.99–2.71)	0.054
Edmondson stage (III-IV vs. I-II)	2.15 (1.31–3.52)	0.003	1.75 (1.05–2.93)	0.032
CD146 (High vs. LOW)	3.64 (2.20–6.03)	<0.001	2.49 (2.43–4.00)	0.001

*Abbreviations: HBsAg* Hepatitis B surface antigen, *AFP* α-fetoprotein

Patients in CD146 high group had obvious higher recurrence probability and shorter overall survival time than those in CD146 low group (Fig. 7a and b). This finding enrolled CD146 with clinical significance. Targeting CD146 may become a potential therapeutic strategy of HCC treatment.

## Discussion

In this study, we demonstrate for the first time that CD146 promotes HCC progression. We confirmed this finding by providing the following evidence. First, CD146 was frequently upregulated in human HCC tissues compared with adjacent noncancerous tissues and a high CD146 level predicted high recurrence probability and poor overall survival time. Second, in vitro experiments showed that overexpression of CD146 in low CD146-expressing HCC cell lines markedly promoted cell migration and invasion whereas interfering with CD146 expression in high CD146-expressing HCC cell lines showed opposite results. In vivo experiments using the orthotropic tumor metastasis model confirmed these results; more intraliver invasion nodules and lung metastatic foci were observed with CD146 overexpressing cell lines and the opposite effects were observed with CD146 silenced cell lines.

We further investigated the potential mechanisms of CD146-induced migration and invasion by whole genome DNA microarrays and WB validation. We found that STAT1 was downregulated whereas IL-8 was upregulated after CD146 overexpression. Although a few reports indicated STAT1 promoted cancer progression [21, 22], it has been widely accepted that STAT1 negatively regulates cancer progression. STAT1 negatively regulates angiogenesis, tumorigenicity, and metastasis of tumor cells by inhibiting the expression of bFGF, MMP-2, and MMP-9 [23]. STAT1 also functions as a suppressor of HCC cell proliferation and a regulator of HCC cell apoptosis by regulating p53 and cyclin E expression [24]. IL-8 was first identified as a chemotactic factor for leukocytes and was demonstrated to function in cancer progression over recent decades. Serum IL-8 was found upregulated in HCC patients and was correlated with larger tumor volume and advanced tumor stage. IL-8 promoted HCC invasion and the incidence of microscopic vessel invasion was significantly higher in IL-8-positive than in IL-8-negative HCC tissues. Further investigation indicated that IL-8 may function by promoting EMT [25–27]. In this study, STAT1 was downregulated and IL-8 was upregulated after CD146 overexpression. Because previous studies showed that STAT1 and IL-8 could regulate HCC metastasis, together these data indicate that CD146 could promote HCC metastasis by, or at least partially by, regulating STAT1 and IL-8. However, the precise mechanism of the regulation process still needs further investigation.

Second, CD146 was reported to promote metastasis by regulating EMT in other solid tumors such as breast tumor [13, 28]. EMT was believed to endow cancer cells with migratory and invasive properties and induce cancer stem cell properties [29, 30]. However, whether CD146 could induce EMT in HCC has never been explored. We found that CD146 induced E-cadherin loss and vimentin and slug overexpression, and this phenomenon reversed after CD146 inhibition. This indicated that CD146-induced EMT may also count for CD146-induced metastasis of HCC cell lines.

Last, we investigated the potential signaling pathway of CD146-induced metastasis. According to Gene Ontology analysis, a MAPK1-associated signaling pathway was indicated to play a central role. We confirmed that MAPK1 was phosphorylated after CD146 overexpression and the opposite effects were observed after CD146 downregulation. MAPKs are generally expressed in all cell types and transduce stimulations, growth factors, cytokines, and extracellular stress signals into intracellular responses [31]. MAPK1 is one of the subfamilies of MAPKs that functions in various physiological and pathological processes. The role of MAPK1 in cancer development has also been widely explored. MAPK1 phosphorylation was involved in S100p-induced proliferation and metastasis, as well as EMT in colon cancer [32]. The insulin-like growth factor binding protein 5 inhibited MAPK1, resulting in compromised growth and migration ability in melanoma cells [33]. Scribble acts as an oncogene in Eμ-myc-driven lymphoma, and the potential mechanism partially involves the activation of MAPK1 [34]. In HCC development, simultaneous activation of the MAPK1 pathways has been shown to enhance cell-cycle progression [35]. Recently, EDIL3 was identified to promote angiogenesis, metastasis, and recurrence of HCC by MAPK1 and TGF-β signaling pathways [36]. These studies indicate the pivotal role of MAPK1 in cancer progression, especially in cancer metastasis and EMT.

## Conclusion

In conclusion, our findings show that CD146 promotes migration and invasion of HCC cell lines by regulating EMT. CD146-induced IL-8 upregulation and further activation of MAPK1 signaling pathways may be its potential regulating methods. However, unraveling the detailed signaling pathways involved in CD146-induced EMT will require further research. In recent years, circulating cancer cell research has Enrich the means to detect tumor cells [37, 38], and the potential of CD146 on circulating tumor cell research still need further investigation.

To our knowledge, this is the first report to identify the striking correlation between high CD146 expression and poor prognosis of HCC patients. Furthermore, we demonstrated that CD146 plays a critical role in HCC progression by MAPK1 signaling dependent EMT activation. Our results indicate that CD146 can be used as a potential HCC marker and may therefore be helpful in developing an effective treatment against cancer.

## Additional files

Additional file 1: Table S1. Primer pairs used for real-time PCR in this study. (DOCX 12 kb) Additional file 2: Table S2. Antibodies used in this study. (DOCX 13 kb) Additional file 3: Table S3. CD146 interfere sequence used in this study. (DOCX 12 kb) Additional file 4: Figure S1. Western Blotting of 17 paired HCC and peritumor tissues. (TIF 6418 kb) Additional file 5: Figure S2. CD146 expression doesn’t impact cell proliferation in 97H or 97 L cell lines. (TIF 5396 kb) Additional file 6: Figure S3. CD146 expression doesn’t impact apoptosis and cell cycle in 97H or 97 L cell lines. (TIF 6329 kb) Additional file 7: Table S4. Cancer related pathway genes modulated after CD146 upregulation. (DOCX 13 kb)

## Abbreviations

EMT: epithelial mesenchymal transition
ERK1/2: extracellular signal regulated kinase 1 and 2
HCC: hepatocellular carcinoma
IL-8: interleukin-8
MAPK 1: mitogen activated protein kinase 1
RT-PCR: real-time polymerase chain reaction
STAT1: signal transducer and activator of transcription 1
TTR: time to recurrence
WB: western blotting
