# Supplementary material for: CD146 promotes metastasis and predicts poor prognosis of hepatocellular carcinoma
Source: J Exp Clin Cancer Res. 2016 Feb 29;35:38. doi: 10.1186/s13046-016-0313-3 (PMC4772456; doi:10.1186/s13046-016-0313-3)
Supplement: Additional file 1: Table S1. — Primer pairs used for real-time PCR in this study. (DOCX 12 kb) [file 13046_2016_313_MOESM1_ESM.docx]

Supplementary Table 1. Primer pairs used for real-time PCR in this study

| **Gene symbol** | **Sequence 5’-3’** |  | | **Amplicon size** |
| --- | --- | --- | --- | --- |
| **CD146** | F:5'AGGAGCCAAACATCCAGGTCA3' |  |  | |
|  | R:5'GTGTACAAACCACTCGACTCCACAG3' |  | 199bp | |
|  |  |  |  | |
| **Beta actin** | F:5'TTGTTACAGGAAGTCCCTTGCC3' |  |  | |
|  | R:5'ATGCTATCACCTCCCCTGTGTG3' |  | 101bp | |
