# Supplementary material for: CD146 promotes metastasis and predicts poor prognosis of hepatocellular carcinoma
Source: J Exp Clin Cancer Res. 2016 Feb 29;35:38. doi: 10.1186/s13046-016-0313-3 (PMC4772456; doi:10.1186/s13046-016-0313-3)
Supplement: Additional file 2: Table S2. — Antibodies used in this study. (DOCX 13 kb) [file 13046_2016_313_MOESM2_ESM.docx]

Supplementary Table 2. Antibodies used in this study

| **Antibody** | **Manufacturers** |  | | **Application** |
| --- | --- | --- | --- | --- |
| **CD146** | ab75769 abcam, USA |  | 1:1000 | |
| **E-cadherin** | #999 CST,USA |  | 1:1000 | |
| **vimentin** | #7431 CST,USA |  | 1:1000 | |
| **slug** | #6591 CST,USA |  | 1:1000 | |
| **ERK1/2** | #4695 CST,USA |  | 1:1000 | |
| **P-ERK1/2** | #4370 CST,USA |  | 1:1000 | |
| **IL-8** | ab7747 abcam, USA |  | 1:200 | |
| **STAT1** | #9175 CST,USA |  | 1:500 | |
| **GAPDH** | #2597 CST,USA |  | 1:1000 | |
|  |  |  |  | |
|  |  |  |  | |
