# Supplementary material for: CD146 promotes metastasis and predicts poor prognosis of hepatocellular carcinoma
Source: J Exp Clin Cancer Res. 2016 Feb 29;35:38. doi: 10.1186/s13046-016-0313-3 (PMC4772456; doi:10.1186/s13046-016-0313-3)
Supplement: Additional file 3: Table S3. — CD146 interfere sequence used in this study. (DOCX 12 kb) [file 13046_2016_313_MOESM3_ESM.docx]

Supplementary Table 3. CD146 interfere sequence used in this study

| **Gene ID** | **5’** | **stem** | **loop** | **stem** | **3’** |
| --- | --- | --- | --- | --- | --- |
| **MCAM-RNAi-a** | Ccgg | gtGTTGAATCTGTCTTGTGAA | CTCGAG | TTCACAAGACAGATTCAACAC | TTTTTg |
| **MCAM-RNAi-b** | aattcaaaaa | gtGTTGAATCTGTCTTGTGAA | CTCGAG | TTCACAAGACAGATTCAACAC |  |
