# Supplementary figures and images for: CD146 promotes metastasis and predicts poor prognosis of hepatocellular carcinoma
Source: J Exp Clin Cancer Res. 2016 Feb 29;35:38. doi: 10.1186/s13046-016-0313-3 (PMC4772456; doi:10.1186/s13046-016-0313-3)

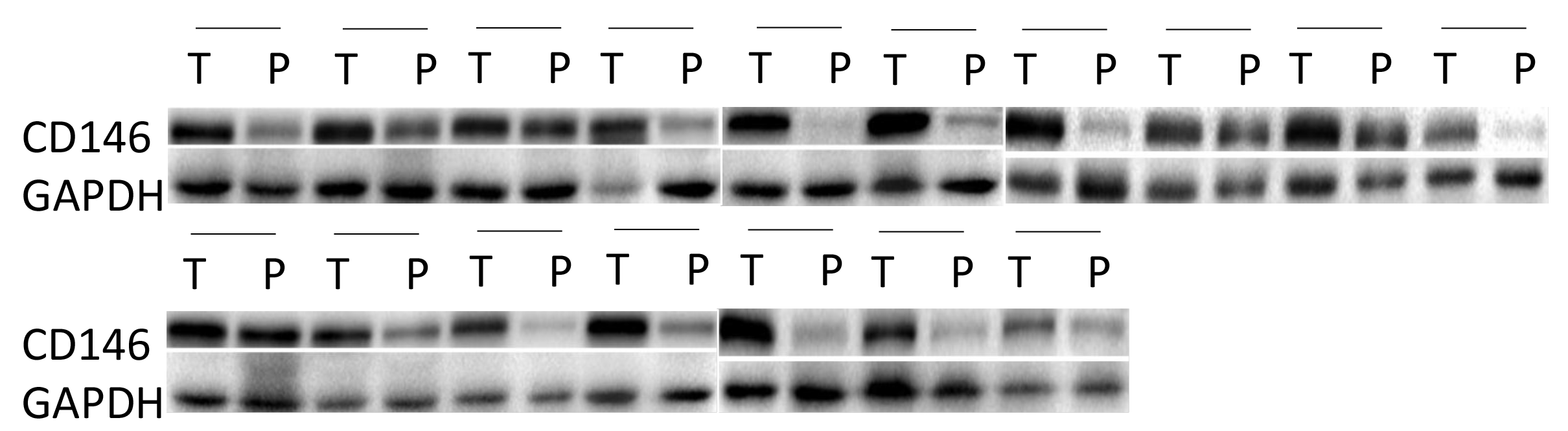

Supplement: Additional file 4: Figure S1. — Western Blotting of 17 paired HCC and peritumor tissues. (TIF 6418 kb) [file 13046_2016_313_MOESM4_ESM.tif]

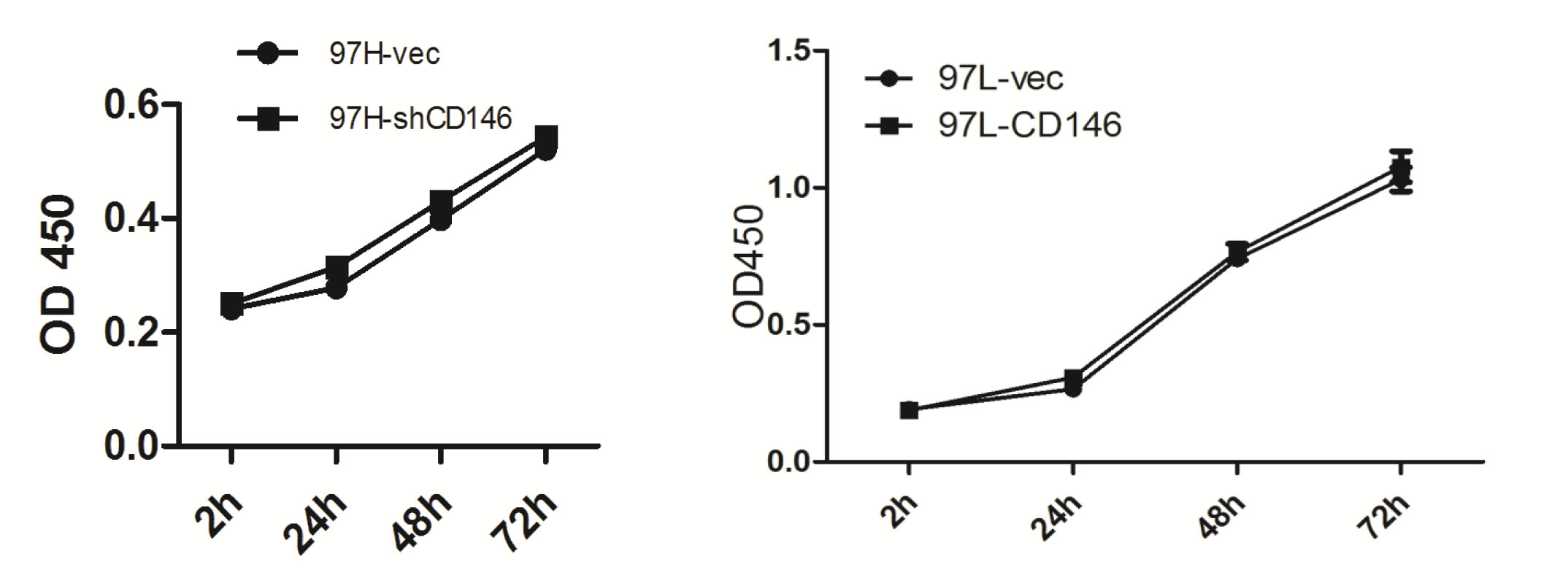

Supplement: Additional file 5: Figure S2. — CD146 expression doesn’t impact cell proliferation in 97H or 97 L cell lines. (TIF 5396 kb) [file 13046_2016_313_MOESM5_ESM.tif]

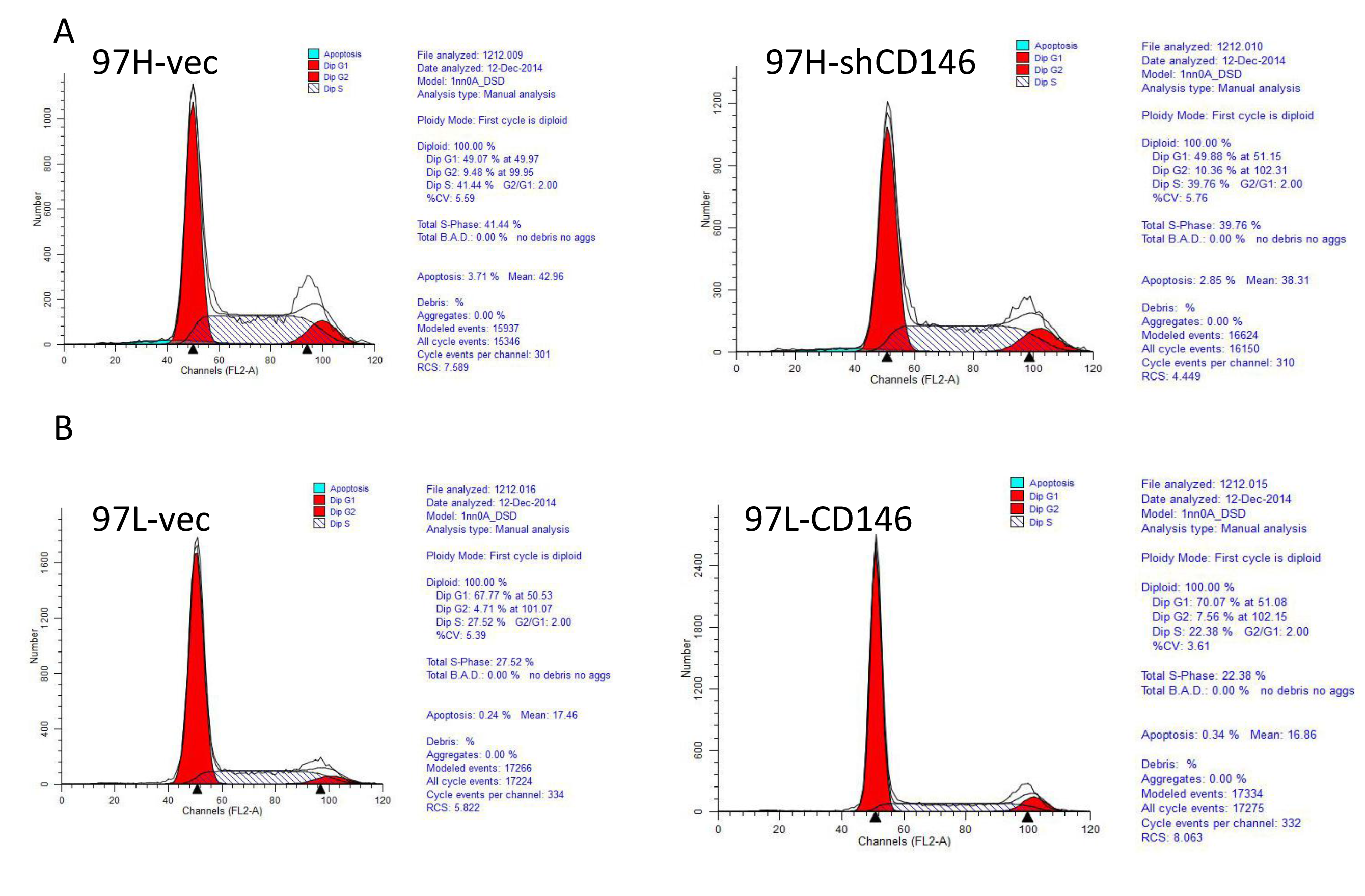

Supplement: Additional file 6: Figure S3. — CD146 expression doesn’t impact apoptosis and cell cycle in 97H or 97 L cell lines. (TIF 6329 kb) [file 13046_2016_313_MOESM6_ESM.tif]
