# Supplementary material for: CD146 promotes metastasis and predicts poor prognosis of hepatocellular carcinoma
Source: J Exp Clin Cancer Res. 2016 Feb 29;35:38. doi: 10.1186/s13046-016-0313-3 (PMC4772456; doi:10.1186/s13046-016-0313-3)
Supplement: Additional file 7: Table S4. — Cancer related pathway genes modulated after CD146 upregulation. (DOCX 13 kb) [file 13046_2016_313_MOESM7_ESM.docx]

Supplementary Table 4. Cancer related pathway genes modulated after CD146 upregulation

|  | **Accesion number** | **Fold change** | ***P* value** |
| --- | --- | --- | --- |
| **CD146** | Gene ID: 4162 | 5.0043163 | 3.08E-05 |
| **EGFR** | Gene ID: 1956 | 1.6952908 | 0.0004037 |
| **CXCL8** | Gene ID: 3576 | 2.1595967 | 0.0000665 |
| **IL6** | Gene ID: 3569 | 2.9572866 | 0.0000005 |
| **KITLG** | Gene ID: 4254 | -1.8453050 | 0.0002115 |
| **LAMA2** | Gene ID: 3908 | -1.5147285 | 0.0000214 |
| **COL4A1** | Gene ID: 1282 | 1.7585322 | 0.0000199 |
| **MMP1** | Gene ID: 4312 | 2.2173114 | 0.0000018 |
| **CCND1** | Gene ID: 595 | 1.8555661 | 0.0000106 |
| **FGF2** | Gene ID: 2247 | 1.5277121 | 0.0000404 |
| **FGF13** | Gene ID: 2258 | -1.8553283 | 0.0007705 |
| **MECOM** | Gene ID: 2122 | -1.7281221 | 0.0018369 |
| **STAT1** | Gene ID: 6772 | -1.5886195 | 0.0000237 |
| **ETS1** | Gene ID: 2113 | 2.4073825 | 0.0000039 |
| **PTGS2** | Gene ID: 5743 | 1.7138067 | 0.0038492 |
| **WNT5A** | Gene ID: 7474 | 1.7316617 | 0.0001902 |
| **TPR** | Gene ID: 7175 | -1.5396863 | 0.0002438 |
